# Surveillance of Cardiovascular Risk Factors in the Fifth Military Sector Health Center, Ngaoundéré, Cameroon: Observational Study

**DOI:** 10.2196/18567

**Published:** 2020-11-26

**Authors:** Williams Bell Ngan, Lawrence Essama Eno Belinga, Alain Serges Patrick Essam Nlo'o, Frederic Roche, Luc Goethals, Samuel Honoré Mandengue, Bienvenu Bongue

**Affiliations:** 1 Department of Military Health Yaoundé Cameroon; 2 Faculty of Science University of Douala Douala Cameroon; 3 Autonomic Nervous System Research Laboratory University of Jean Monnet Saint-Etienne France; 4 Douala General Hospital Douala Cameroon; 5 Faculty of Medicine and Pharmaceutical Sciences University of Douala Douala Cameroon; 6 Support and Education Technic Centre of Health Examination Centres (CETAF) Saint-Etienne France

**Keywords:** prevention, noncommunicable disease, cardiovascular diseases, cardiovascular risk, soldiers

## Abstract

**Background:**

Noncommunicable diseases (NCDs) are the leading causes of death worldwide. They were responsible for 40 million of the 57 million deaths recorded worldwide in 2016. In Cameroon, epidemiological studies have been devoted to NCDs and their risk factors. However, none provides specific information on their extent or the distribution of their risk factors within the Cameroonian defense forces.

**Objective:**

The objective of our study was to assess the cardiovascular risk of a Cameroonian military population compared with that of its neighboring civilian population.

**Methods:**

We conducted a cross-sectional study that involved subjects aged 18 to 58 years, recruited from October 2017 to November 2018 at the Fifth Military Sector Health Center in Ngaoundéré, Cameroon. Data collection and assessment were done according to the World Health Organization (WHO)’s STEPS manual for surveillance of risk factors for chronic NCDs and the Alcohol Use Disorders Identification Test. Five cardiovascular risk factors were assessed: smoking, harmful alcohol consumption, obesity/overweight, hypertension, and diabetes. The risk was considered high in subjects with 3 or more of the factors. Univariate analysis and multivariate logistic regression were carried out according to their indications.

**Results:**

Our study sample of 566 participants included 295 soldiers and 271 civilians of the same age group (median age 32 years versus 33 years, respectively; *P*=.57). The military sample consisted of 31 officers and 264 noncommissioned officers (NCOs). Soldiers were more exposed to behavioral risk factors than civilians, with a prevalence of smoking of 13.9% versus 4.4% (*P*<.001) and excessive alcohol consumption of 61.7% versus 14.8% (*P*<.001). They also presented with a higher cardiovascular risk than civilians (odds ratio 2.7, 95% CI 1.50-4.81; *P*<.001), and among the military participants, the cardiovascular risk was higher for officers than for NCOs (51.6% versus 14.0%, respectively; *P*<.001).

**Conclusions:**

Cameroonian soldiers are particularly exposed to cardiovascular behavioral risk factors and consequently are at higher risk of NCDs.

**Trial Registration:**

ClinicalTrials.gov NCT04315441; https://clinicaltrials.gov/ct2/show/NCT04315441

## Introduction

In the last three decades, infectious diseases such as human immunodeficiency virus/acquired immunodeficiency syndrome and others were the leading causes of mortality in sub-Saharan countries. However, with increased life expectancy and the adoption of harmful lifestyles, noncommunicable diseases (NCDs) have emerged among the leading causes of morbidity as well as early death in these countries.

NCDs were responsible for 41 million of the 57 million deaths recorded worldwide in 2016 [1,2]. Low- and middle-income countries were affected by 28 million of these deaths, 82% of which were considered premature because they occurred in people younger than 70 years of age [1,2]. The four main NCDs responsible for the deaths were cardiovascular diseases (CVDs), diabetes, cancer, and chronic respiratory disease, with CVDs (ischemic heart disease, ischemic stroke, and peripheral vascular disease) being responsible for the greatest number of deaths. Ischemic heart disease, responsible for 9.4 million deaths, ranked first among the causes, followed by strokes, which were responsible for 5.8 million deaths. Other circulatory diseases, responsible for 1 million deaths, were the tenth leading cause of death worldwide in 2016 [1].

The NCD epidemic is gaining so much momentum that the military population, which seemed to be spared from these pathologies, is as exposed today as the general population, and sometimes even more so, with the consequences of a deleterious effect on their operational capacity and a high rate of absenteeism [3,4]. The effective response to this public health challenge requires prior identification of the level of exposure of populations in order to implement actions to reduce the risk factors. Several nations have therefore assessed the extent of NCDs in their military populations [3-8].

In Cameroon, epidemiological studies have been devoted to NCDs and their risk factors [9-15]. However, none of them provides specific information on their extent and/or the distribution of their risk factors within its defense forces.

Thus, the objective of our study was to assess the cardiovascular risk of a Cameroonian military population compared with that of a civilian population.

## Methods

### Study Design

We carried out a descriptive and analytical cross-sectional study.

### Study Area and Population

The study involved the soldiers of the Ngaoundéré garrison and the neighboring civilian population. Ngaoundéré is the capital city of the Adamawa Region of Cameroon, with 298,016 inhabitants and a surface area of 62,000 km^2^.

Participation in the study was proposed to all soldiers who came to the Ngaoundéré Fifth Military Sector Medical Center for the annual re-engagement medical visit during the period from January 1, 2017, to November 13, 2018. For inclusion in the study, the participant had to consent and be between 18 and 58 years of age—representing the ages for enrollment and retirement, respectively, for Cameroonian soldiers. The civilian populations were recruited during two free cardiovascular risk factor screening campaigns carried out from January 2017 to November 2018. These campaigns were also open to soldiers who wished to participate.

### Medical Examination, Data Collection, and Definition of Variables  

A questionnaire, developed from the World Health Organization (WHO)’s STEPS Manual for surveillance of risk factors of NCDs, made it possible to collect sociodemographic information (eg, age, gender, and military rank), information on habits related to healthy living (alcohol consumption and smoking in particular), and information on the medical history of study participants. An additional tool—the Alcohol Use Disorders Identification Test (AUDIT), developed by the WHO—was used to assess the participant’s level of alcohol consumption [16]. Anthropometric data such as height and weight were collected to determine BMI.

Blood pressure was measured using an electronic blood pressure monitor. The individual was seated and rested for 15 minutes before a measurement was taken on each arm with a 10-minute interval between measurements. For those with diastolic and/or systolic blood pressures higher than or equal to 90 mmHg and/or 140 mmHg, respectively, a second measurement was taken on both arms with a 10-minute interval between measurements. In all cases, the lower values were retained for each participant.

The fasting capillary blood glucose test was carried out using a strip and a glucometer.

The variables defined from the questionnaire and measurements taken were as follows:

- smoking: “Yes” response in answer to the question, “Do you smoke?”
- alcohol intake: “Yes” response in answer to the question, “Do you consume alcohol?”
- weight status: 4 categories were defined according to BMI: underweight (BMI<18.5 kg/m^2^), normal weight (18≤BMI<25 kg/m²), overweight (25≤BMI<30 kg/m²), and obese (BMI≥30 kg/m²)
- high blood pressure (hypertension): defined as having a systolic blood pressure ≥140 mmHg and/or diastolic blood pressure ≥90 mmHg (BP-103H Upper Arm Lifestyle Blood Pressure Monitor, Idass) or taking any medication prescribed by medical personnel to reduce hypertension
- diabetes: defined as having a fasting blood glucose ≥7 mmol/L (OneTouch Ultra 2, Lifescan Canada Ltd) or taking any medication prescribed by medical personnel to treat diabetes
- cardiovascular risk: determined by summing up the individual’s risk factors, including being a smoker, excessive alcohol consumption (defined as an AUDIT score ≥8), being overweight or obese, or having hypertension or diabetes. It was considered high if the participant had a combination of at least 3 risk factors.

### Statistical Analyses

All analyses were performed using Epi Info software (version 7.2; Centers for Disease Control and Prevention). The Pearson chi-square test was used for comparing proportions. The Mann-Whitney test and analysis of variance were carried out according to their indications to compare the numeric variables. A multiple logistic regression analysis was performed to determine the population with the greatest cardiovascular risk regardless of age or gender. The significance threshold was set at *P*<.05.

### Ethical Considerations

The study was authorized by the military hierarchy (ref 016082/AU/DSM/RSM3/SSM5), and the participants all agreed to the use of the information collected for the purposes of this study.

## Results

We sampled 566 volunteers comprised of 295 soldiers and 271 civilians of approximately the same ages (mean 35 [SD 11] years versus 36 [SD 11] years, respectively; *P*<.57). A total of 460 of 566 (81.3%) people in the sample population were men. The BMI of the military personnel was on average higher than that of their civilian counterparts (mean 26.18 [SD 3.96] kg/m^2^ versus 24.74 [SD 4.91] kg/m^2^; *P*<.001). On the other hand, clinical parameters such as blood glucose and blood pressure levels were similar for the two populations. The demographic characteristics and clinical parameters are presented in Table 1.

**Table 1:** Demographic characteristics and clinical parameters of the sample population.

Characteristics		Total sample (n=566)	Military (n=295)	Civilians (n=271)	*P* value
**Gender, n (%)**					
	Women	105 (18.7)	34 (11.5)	72 (26.6)	.001^a^
	Men	461 (81.3)	261 (88.5)	199 (73.4)	
Age (years), median (IQR)		33 (26-44)	32 (26-43)	33 (26-45)	.65^b^
BMI (kg/m^2^), median (IQR)		25.20 (22.49-27.97)	25.73 (23.72-28.04)	24.22 (21.20-27.43)	.001^b^
SBP^c^ (mmHg), median (IQR)		126 (116-136)	127 (117-137)	125 (114-135)	.12^b^
DBP^d^ (mmHg), median (IQR)		77 (69-86)	78 (69-87)	77 (69-85)	.19^b^
Glycemia (mmol/L), median (IQR)		5.29 (4.89-5.79)	5.19 (4.88-5.73)	5.36 (4.95-5.79)	.47^b^

^a^Calculated using chi-square test.

^b^Calculated using Mann-Whitney test.

^c^SBP: systolic blood pressure.

^d^DBP: diastolic blood pressure.

Of the 295 military participants, 31 (10.5%) were officers and 264 (89.5%) were noncommissioned officers (NCOs), with the former being older than the latter (mean age 50 [SD 8] years versus 34 [SD 10] years; *P*<.001).

With respect to the distribution of cardiovascular risk factors, the number per participant was higher among the military population than among the civilians (median [IQR]: 2 [1-2] versus 1 [0-1]; *P*<.001). There were 279 (279/566, 49.3%) people who reported consuming alcohol. Among them, the proportion of soldiers was greater than that of civilians (70.2% versus 26.6%, respectively; *P*<.001). The AUDIT scores were higher in the military population than in the civilian population (median [IQR]: 9 [0-9] versus 0 [0-3], respectively; *P*<.001). Even when the analysis incorporated nonmodifiable cardiovascular risk factors, such as age and gender, the military population still had a higher risk profile than the civilians (odds ratio [OR] 2.7, 95% CI 1.51-4.81). Tables 2–4 show the distribution of the risk factors in the sample population.

In the military population, officers had significantly more cardiovascular risk factors than NCOs (OR 6.54, 95% CI 2.98-14.35). This is shown in Table 5.

**Table 2:** Distribution of cardiovascular risk factors in the sample population.

Risk factors	Total sample (N=566), n (%)	Military (n=295), n (%)	Civilians (n=271), n (%)	*P* value
Smoking	53 (9.4)	41 (13.9)	12 (4.4)	.001
Excessive alcohol consumption	222 (39.2)	182 (61.7)	40 (14.8)	.001
Diabetes	19 (3.4)	8 (2.7)	11 (4.1)	.37
Obesity or overweight	292 (51.6)	177 (60.0)	115 (42.4)	.001
Blood pressure above normal	132 (23.3)	68 (23.1)	64 (23.6)	.87
High cardiovascular risk	77 (13.6)	53 (18.0)	24 (8.9)	.001

**Table 3:** Distribution of cardiovascular risk factors by gender.

Risk factors	Male			Female		
	Military (n=295), n (%)	Civilians (n=271), n (%)	*P* value	Military (n=295), n (%)	Civilians (n=271), n (%)	*P* value
Smoking	40 (15.3)	12 (6.0)	.002	1 (2.9)	0 (0)	.32
Excessive alcohol consumption	165 (63.2)	34 (17.1)	.001	17 (50.0)	6 (8.3)	.001
Diabetes	7 (2.7)	10 (5.0)	.19	1 (2.9)	1 (1.4)	.058
Obesity or overweight	151 (57.9)	74 (37.2)	.001	26 (76.5)	41 (56.9)	.05
Blood pressure above normal	60 (23.0)	43 (21.6)	.72	8 (23.5)	21 (29.2)	.54
High cardiovascular risk	49 (18.8)	19 (9.6)	.01	4 (11.8)	5 (6.9)	.41

**Table 4:** Multivariate analysis of the relationship between high cardiovascular risk, occupation, age, and gender.

Variables	Odds ratio (95% CI)	*P* value
Age	1.11 (1.08-1.13)	0.001
Gender (male/female)	1.36 (0.61-3.03)	0.45
Occupation (military/civilian)	2.70 (1.51-4.81)	0.001

**Table 5:** Distribution of cardiovascular risk factors in the military population.

Risk factors	Officers (n=31), n (%)	Noncommissioned officers (n=264), n (%)	*P* value^a^
Smoking	8 (25.8)	33 (12.5)	.04
Excessive alcohol consumption	24 (77.4)	158 (59.9)	.06
Diabetes	2 (6.5)	6 (2.3)	.17
Obesity or overweight	26 (83.9)	151 (57.2)	.001
Blood pressure above normal	17 (54.9)	51 (19.3)	.001
High cardiovascular risk	16 (51.6)	37 (14.0)	.001

^a^Calculated using chi-square test.

## Discussion

### Principal Findings

The objective of this study was to determine the level of exposure of the military population of Ngaoundéré to CVD. Our results suggested that they were effectively at risk and that the risk factors were generally more frequent among them than in the civilian population. In fact, the number of risk factors in the military population was double that in the civilian population. Similarly, behavioral cardiovascular risk factors such as smoking and excessive alcohol consumption were observed in more military participants than in civilians. Within the Cameroonian military population, we found that officers had a higher cardiovascular risk than NCOs, which was hardly surprising, given the fact that the officers were significantly older.

Considering the general distribution of these cardiovascular risk factors in our sample population, there was barely a difference compared with the prevalence observed in other studies in Cameroon. For hypertension, we found a prevalence of 23.3%, which was obviously different from the current general prevalence in Cameroon that is estimated at 32.1% [17]. However, it should be noted that at least one-half of our participants were younger than 33 years of age, and the prevalence of hypertension among those under 35 years of age in Cameroon is 24% [17], which is perfectly in line with our result of 23.3%. In addition, the proportion of patients with hypertension in our study was in agreement with the 20.43% found in a study carried out in 2016 in the general population of Ngaoundéré [15]. In our study, the proportion of smokers was 9.4%, which was very close to the prevalences of 8.4% and 8.3% observed by other authors in the cities of Yaoundé [13] and Ngaoundéré [15], respectively. Diabetes was present in 3.4% of our participants, which was not far off the prevalence of 5.8% found in a recent study in Cameroon [14].

With regard to the distribution of cardiovascular risk factors among the military population, our overall results showed some similarities with studies carried out in other countries. Thus, our finding of 23.1% of soldiers with hypertension was not far off the 28.4% found in the Senegalese army [4] or the 21.7% in the Guinea-Conakry army [8].

On the contrary, in industrialized countries such as the United States, the prevalence of hypertension is 13% [3]. This difference can be explained by the fact that many industrialized nations embarked on the fight against NCDs in their armies much earlier than African countries did [3]. While 13.9% of Cameroonian soldiers used tobacco, other armies had higher prevalences, such as 17.3% in Senegal, 47.3% in Guinea-Conakry, 20.3% in Nigeria, 34.8% in Uganda, 47.1% in Côte d’Ivoire, and 32% in Taiwan [4,18-22]. This difference can be explained by the fact that in general, the Cameroonian population, including the military population, seems to have a low propensity for smoking [13-15]. Also, the proportion of Cameroonian soldiers who were obese or overweight was 60%, which was very similar to the proportion observed in Saudi Arabia (69.9%) [8].

### Study Limitations

Some limitations of our study on the Cameroonian defense forces were that these results would have been more complete if they had included other cardiovascular risk factors such as hyperlipidemia/hypercholesterolemia, food imbalance, and physical inactivity. However, the assessment of physical activity was deliberately excluded from the procedures because the practice of sports and other types of maneuvers are an integral part of the lives of Cameroonian soldiers. For example, 2 days of collective sports per week are the compulsory minimum for all military units. Therefore, our hypothesis was that, a priori, Cameroonian soldiers carry out regular physical activity.

The lipid and cholesterol levels of the participants were not evaluated due to our limited financial means. Nonetheless, some authors suggest that the BMI assessment is an alternative measure that can be substituted for the cholesterol assessment in estimating cardiovascular risk in low-income countries [23,24]. It is also important to put BMI into perspective in participants, such as military personnel, who do a lot of sports because BMI can be high in athletes. This is because muscle mass represents a significant weight that does not necessarily correspond to body fat. Therefore, BMI can certainly overestimate the weight of participants, although it remains a very practical epidemiological tool for the evaluation of weight status. It gives an overview, which can be estimated more precisely with other specific tools.

We were unable to include the evaluation of the participants’ diets because during the pretesting of the questionnaire used in our study, people found it difficult to respond clearly to the questions about the amounts and types of food they consumed.

Among the limitations of the study, we should also mention the sample size. However, this was an observational study from which we could show differences—for example, in the use of tobacco. Furthermore, the population of the Ngaoundéré area is not representative of the Cameroonian population, either in soldiers or in civilians, because this city is close to an armed conflict area. As a result, the soldiers in this garrison could be selectively those who are more able to fight, while the civilians could include many internally displaced persons fleeing from the looting and killing by insurgents in their villages.

Finally, the WHO’s STEPS methodology is designed to provide standardized information on key modifiable risk factors that can be measured in population-based surveys without the need for high-technology instruments. However, since the behavioral risk factors were self-reported, some of the information could have been concealed, especially those related to alcohol and tobacco use.

We chose to sum up of the cardiovascular risk factors because scoring methods are often used in studies conducted on non-African populations, and their applicability to African subjects is much discussed. An alternative would have been the use of the WHO/International Society of Hypertension risk prediction charts, but these tables are intended for subjects aged 40 years and older [25,26]. Therefore, we opted for this simpler method already used by other authors [4].

Despite these shortcomings, which can be remedied in future studies, our results remain valid and provide information on the distribution of cardiovascular risk factors in the Cameroonian Armed Forces. They can easily be taken into account when developing strategies for the reduction of these risk factors in the country of Cameroon in general, and in the Cameroonian defense forces in particular.

### Conclusion

Cameroonian soldiers are particularly exposed to cardiovascular behavioral risk factors, which implies that national programs in the fight against NCDs and their risk factors should devote greater attention to the military population. These programs should focus on providing adequate sensitization on complications arising from CVDs.

## Abbreviations

AUDIT: Alcohol Use Disorders Identification Test
CVD: cardiovascular disease
NCD: noncommunicable disease
NCO: noncommissioned officer
WHO: World Health Organization
